# Exploring regression to the mean in visual acuities by investigating measurements at two consecutive time points in untreated fellow eyes

**DOI:** 10.1038/s41433-022-02349-z

**Published:** 2022-12-10

**Authors:** Ella Preston, Robin D. Hamilton, Omar A. Mahroo

**Affiliations:** 1 Retinal Service, Moorfields Eye Hospital, London, UK; 2 Institute of Ophthalmology, University College London, Bath Street, London, UK; 3 Section of Ophthalmology, King’s College London, St Thomas’ Hospital Campus, London, UK

**Keywords:** Prognosis, Retina

In clinical trials and real-world data, eyes with worse baseline vision frequently demonstrate greater visual benefit [1–3]. This can be attributed to the “ceiling effect” of higher visual acuities (with less room for improvement). Sometimes, an observed decline in acuity in those with better baseline vision is taken to support earlier intervention. However, “regression to the mean” is also likely to contribute: in any measurement having variability (including measurement error or fluctuations in biological state), more extreme initial values are likely, on subsequent measurements, to be closer to the mean. We investigated this effect by analysing visual acuity measurements in untreated fellow eyes.

We explored visual acuity measurements in untreated eyes, comparing changes in the top and bottom quintile visual acuities at consecutive visits, both forwards and backwards in time. Measurements from patients undergoing unilateral antiVEGF treatment (for neovascular AMD or for retinal vein occlusion macular oedema) in the intravitreal injection services of the authors were included. The retrospective database search covered a 5.5 year period. For each patient, acuities at the first two consecutive time points (providing these were ≤90 days) were included, excluding those with visual acuities <10 ETDRS letters at either visit. Mean acuities for the top 20% and bottom 20% at Visit 1 were compared with those at Visit 2. The same was done for the top and bottom 20% at the second visit (comparing with Visit 1).

1375 patients met the inclusion criteria. Mean (SD) interval between visits was 31.0 (7.7) days (ranging from 3 to 89 days). Mean (SD) acuities overall were 74.3 (15.9) and 74.7 (16.1) letters at first and second visits respectively. For the top quintile (*n* = 275) at Visit 1, mean acuity was lower at Visit 2 (mean difference 1.0 letters, *p* < 0.0001); for the bottom quintile at Visit 1, mean visual acuity was higher at Visit 2 (mean difference 2.3 letters, *p* < 0.0001). For the top quintile at Visit 2, mean acuity was lower at Visit 1 (mean difference 1.5 letters, *p* < 0.0001). For the bottom quintile at Visit 2, mean acuity was higher at Visit 1 (mean difference 1.6 letters, *p* = 0.0039). Figure 1 shows mean acuities by quintile.

**Fig. 1:**
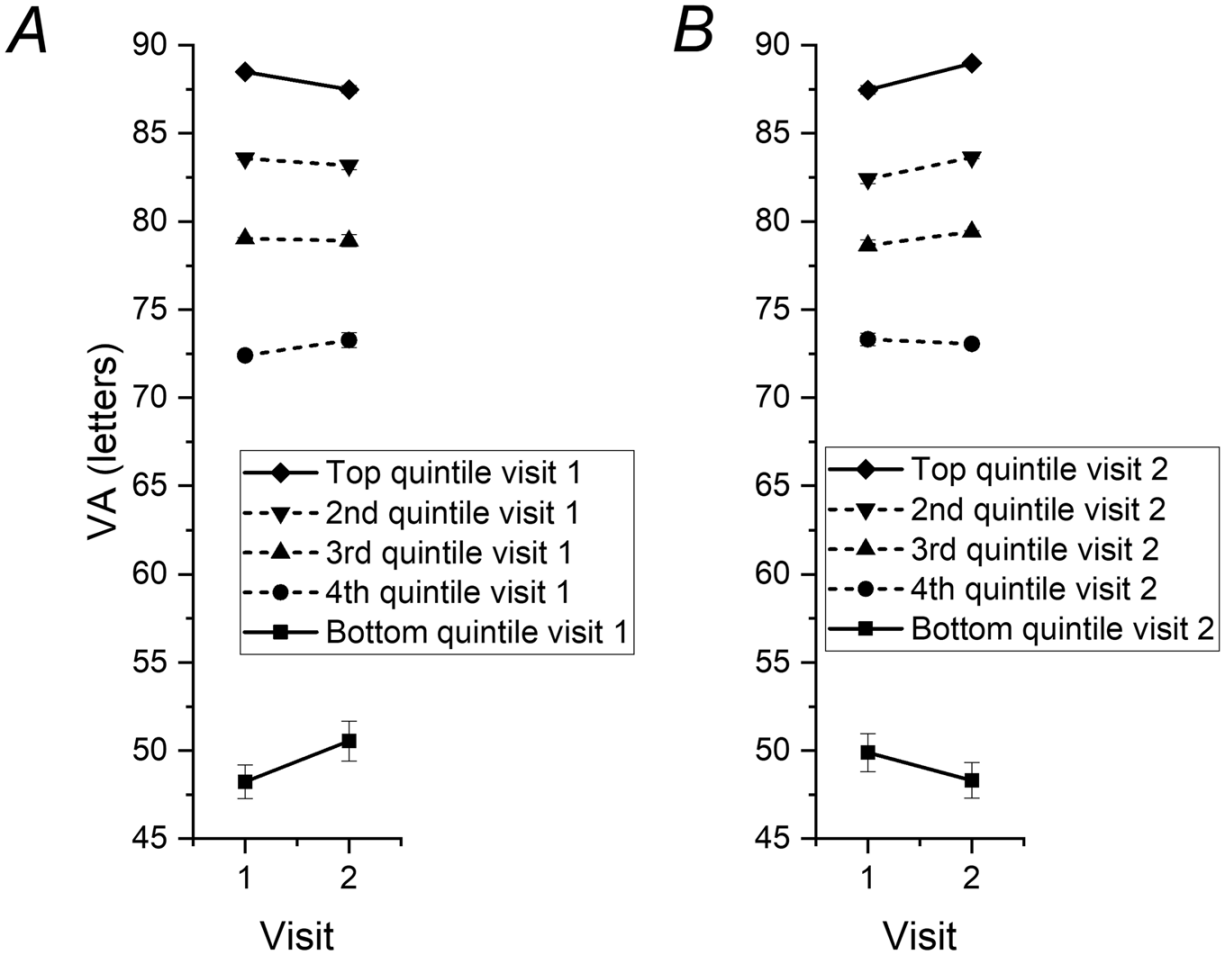
Mean visual acuity measurements by quintile in untreated eyes at two consecutive visits. **A** Points plot mean (±SD) visual acuities at each visit for each quintile defined by visual acuity at Visit 1. **B** Mean (±SD) visual acuities plotted by quintile as in (**A**), but quintiles are defined by acuity at Visit 2. The phenomenon of regression to the mean is observed in both cases: for top quintiles at each visit, mean visual acuity was lower at the other visit; for bottom quintiles at each visit, mean acuity was better at the other visit.

The phenomenon of regression to the mean was thus observed. In eyes undergoing no intervention, those in the top quintile appeared to lose vision, whilst those in the lowest quintile appeared to gain vision. This was regardless of whether one looked forwards or backwards in time, and the difference achieved strong apparent statistical significance. However, the effect was small (between 1 and 2.3 letters) in our cohort. Nevertheless, we recommend that the phenomenon should be considered whenever comparing outcomes in subgroups that have been stratified by baseline values of the same parameter. In eyes with active disease, where there is often greater variability or fluctuation in vision, the effect might be greater. The regression to the mean effect has also been reported in laser refractive surgery [4] and as an explanation for why a simulated switch between different antiVEGF agents could appear to bring about visual gains [5, 6].

## Data Availability

The data processed for the analysis in this manuscript are available on request.
